# Evidence for a role of glutamate as an efferent transmitter in taste buds

**DOI:** 10.1186/1471-2202-11-77

**Published:** 2010-06-21

**Authors:** Aurelie Vandenbeuch, Marco Tizzano, Catherine B Anderson, Leslie M Stone, Daniel Goldberg, Sue C Kinnamon

**Affiliations:** 1 Department of Otolaryngology, University of Colorado, Denver, CO, USA; 2 Department of Cell and Development Biology, University of Colorado, Denver, CO, USA; 3 Department of Biomedical Sciences, Colorado State University, Fort Collins, CO, USA; 4 Rocky Mountain Taste and Smell Center, University of Colorado, Denver, CO, USA

## Abstract

**Background:**

Glutamate has been proposed as a transmitter in the peripheral taste system in addition to its well-documented role as an umami taste stimulus. Evidence for a role as a transmitter includes the presence of ionotropic glutamate receptors in nerve fibers and taste cells, as well as the expression of the glutamate transporter GLAST in Type I taste cells. However, the source and targets of glutamate in lingual tissue are unclear. In the present study, we used molecular, physiological and immunohistochemical methods to investigate the origin of glutamate as well as the targeted receptors in taste buds.

**Results:**

Using molecular and immunohistochemical techniques, we show that the vesicular transporters for glutamate, VGLUT 1 and 2, but not VGLUT3, are expressed in the nerve fibers surrounding taste buds but likely not in taste cells themselves. Further, we show that P2X2, a specific marker for gustatory but not trigeminal fibers, co-localizes with VGLUT2, suggesting the VGLUT-expressing nerve fibers are of gustatory origin. Calcium imaging indicates that GAD67-GFP Type III taste cells, but not T1R3-GFP Type II cells, respond to glutamate at concentrations expected for a glutamate transmitter, and further, that these responses are partially blocked by NBQX, a specific AMPA/Kainate receptor antagonist. RT-PCR and immunohistochemistry confirm the presence of the Kainate receptor GluR7 in Type III taste cells, suggesting it may be a target of glutamate released from gustatory nerve fibers.

**Conclusions:**

Taken together, the results suggest that glutamate may be released from gustatory nerve fibers using a vesicular mechanism to modulate Type III taste cells via GluR7.

## Background

L-glutamate (hereafter referred to as glutamate) has been proposed to play a role in neurotransmission in the peripheral taste system [1,2]. Evidence supporting a role for glutamate as a transmitter includes the expression of glutamate receptors in taste cells [3-8] as well as the presence of the glutamate transporter GLAST [9]. However, the origin of glutamate and its sites of action in the taste bud are not well understood. For example, glutamate could be released from taste cells to activate glutamate receptors on adjacent taste cells or afferent nerve fibers. Alternatively, glutamate could be released from either gustatory or somatosensory nerve fibers to modulate the activity of taste cells. Studies examining the function of glutamate as a transmitter in the taste system are complicated by the fact that glutamate is also a taste stimulus that elicits the umami taste (for review, [10-12]. However, gustatory nerve responses to glutamate induced umami taste require much higher concentrations (e.g., 100 mM) than those typically needed to activate neurotransmitter receptors (high μM to low mM) [13,14].

Glutamate, the major excitatory neurotransmitter in the central nervous system of vertebrates, acts on both ionotropic (iGluRs) and metabotropic (mGluR) receptors. Three pharmacological subtypes of iGluRs are activated by glutamate: N-methyl-D-aspartate (NMDA), (S)-2-amino-3-(3-hydroxy-5-methyl-4-isoxazolyl) propionic acid (AMPA) and Kainate receptors. Of these, taste receptor cells express both NMDA and Kainate receptors, which are believed to respond to glutamate as a transmitter rather than a taste stimulus [4,5,15-17], although the receptor subtypes and their expression patterns are not clear. In addition to iGluRs, the peripheral taste system contains several mGluRs, including mGluR1 [8,18], mGluR2 and mGluR3 [6], and mGluR4 [3,7]. These receptors, as well as the heterodimeric umami taste receptor T1R1 + T1R3 likely play a role in the detection of L-glutamate as a tastant, while the mGluRs could also potentially play a role in taste modulation by glutamate released as a transmitter by taste cells or nerve fibers.

Taste cells are aggregated in taste buds, which contain approximately 50-100 individual cells. Generally, 3 types of taste cells are recognized based on functional and morphological markers. Type I cells (about 50% of the total number of cells in a bud) share many features of glial cells in the nervous system [19]. These cells express enzymes for inactivation and uptake of transmitters [20], including the glutamate transporter GLAST [9]. Type II cells, also called "receptor cells" (about 35% of the cells) possess the G protein-coupled taste receptors and machinery for the transduction of sweet, bitter and umami compounds. These cells express downstream signalling effectors such as Gα-gustducin, PLCβ2, IP3R3 and TRPM5 [21,22]. Although they do not form classical synaptic contacts with gustatory nerve fibers, Type II cells release ATP via hemichannels to communicate with nerve endings and adjacent taste cells [23-25]. Finally, Type III cells, also called "synaptic cells" (about 10-15% of the cells) form conventional synapses with afferent gustatory nerve fibers [21,22,26], although the identity of the transmitter at the synapse is not known. Type III cells contain and release serotonin [27-29] and norepinephrine [30], but also express GAD67, an enzyme involved in the biosynthesis of GABA [31]. In this study, we sought evidence for release and function of glutamate as a transmitter in the taste bud.

The participation of glutamate as a neurotransmitter would imply the presence of vesicular transporters for glutamate (VGLUTs), which are responsible for the transport of glutamate from the cytosol into synaptic vesicles. VGLUTs comprise 3 isoforms, VGLUT1, 2 and 3, which are highly expressed in all glutamatergic neurons in the central nervous system (for a recent review, see [32]). In addition, VGLUT3 is expressed in inner hair cells of the cochlea, where it is necessary for activation of auditory afferent neurons [33]. VGLUTs would also imply the presence of glutamate receptors on either nerve fibers or taste cells that are activated by concentrations of glutamate similar to those in the central nervous system. To address these questions, we used a combination of physiological, molecular and immunohistological techniques to show that VGLUTs are not detected in taste cells, but VGLUT1 and 2 are expressed in the afferent nerve fibers surrounding taste cells. Further, we show that the Kainate receptor GluR7 is selectively expressed in Type III taste cells, where it may respond to glutamate released from afferent nerve terminals. Portions of these results were recently presented in abstract form [34,35]

## Results

### Expression of vesicular glutamate transporters (VGLUTs) in taste tissue

**Figure 1:**
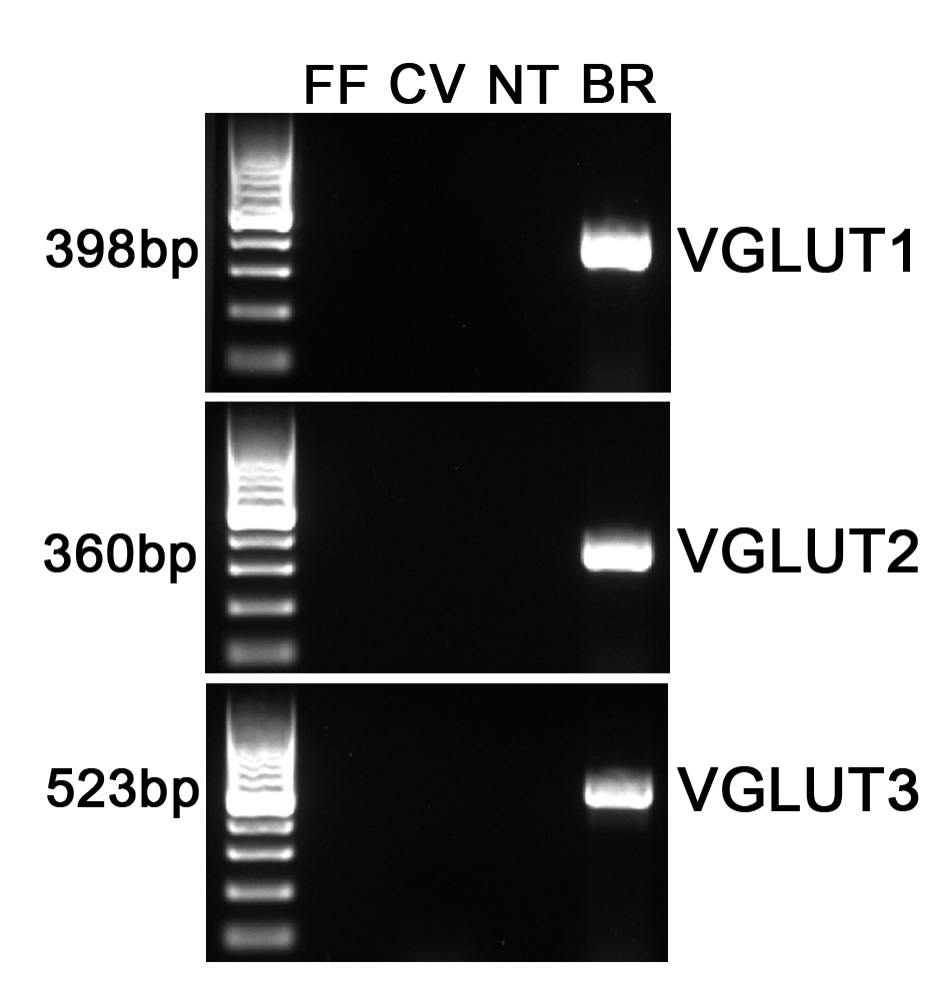
**Gel image of PCR products for VGLUTs 1, 2 and 3 in pooled taste buds**. PCR products for VGLUT 1, 2, and 3 were not observed in pooled taste buds from fungiform (FF) and circumvallate (CV) papillae as well as in non taste epithelium (NT). Brain (BR) was used as a control. Bands are for the expected size for VGLUT1 (398 bp), VGLUT2 (360 bp) and VGLUT3 (523 bp). Left lane indicates a 100-bp ladder.

**Figure 2:**
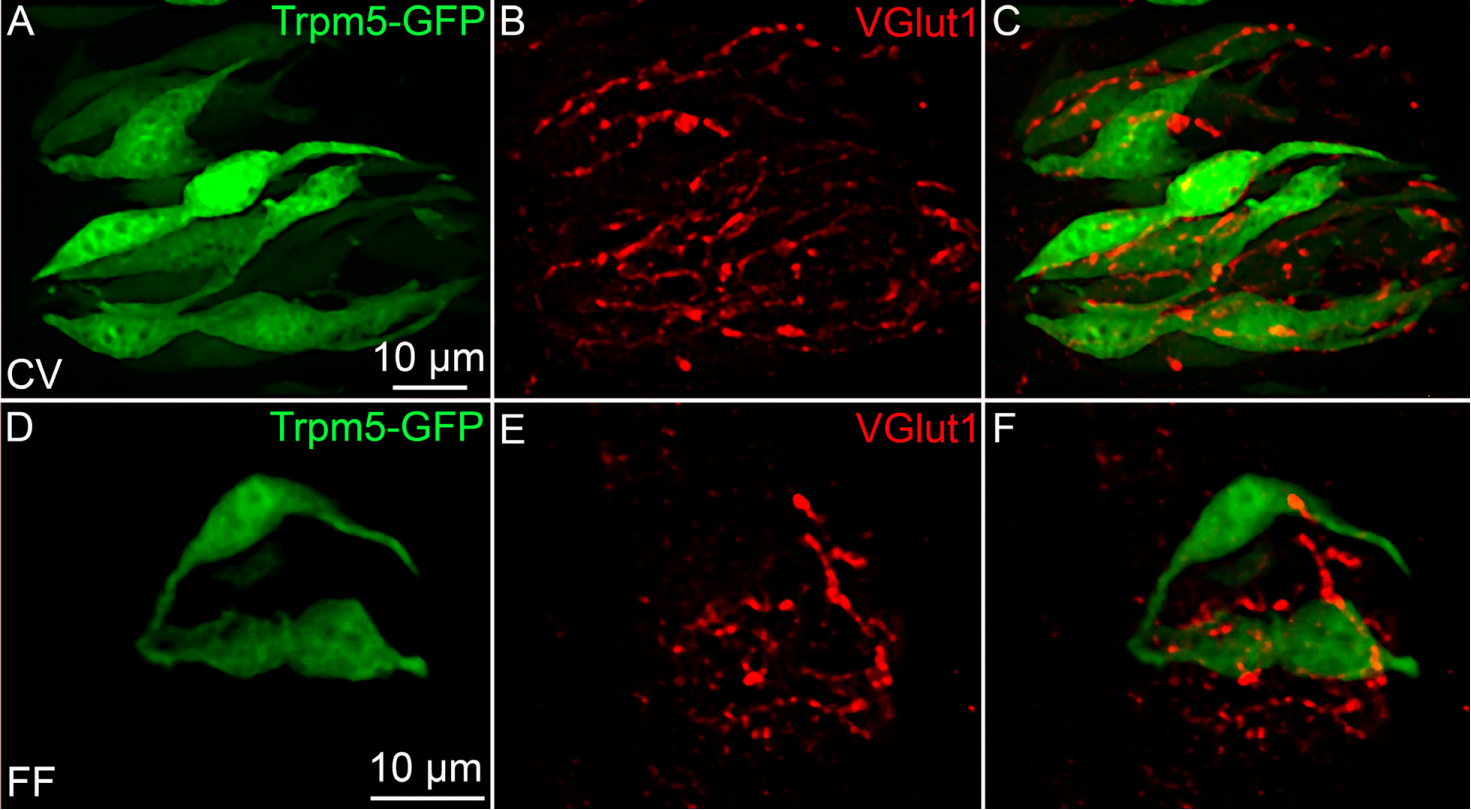
**VGLUT1 immunoreactivity in TRPM5-GFP mice**. The figures illustrate the expression of GFP under the control of the TRPM5 promoter (green), VGLUT1 (red) in circumvallate papillae (CV; A-C), fungiform papillae (FF; D-F). In circumvallate taste buds, VGLUT1 is not expressed in taste cells but only in nerve fibers surrounding these taste cells. Each figure represents merged images from a Z-series. The Photoshop digital filters Dust & Scratches and Unsharp Mask were used in some images to eliminate pixel noise in this and subsequent figures containing confocal images.

**Figure 3:**
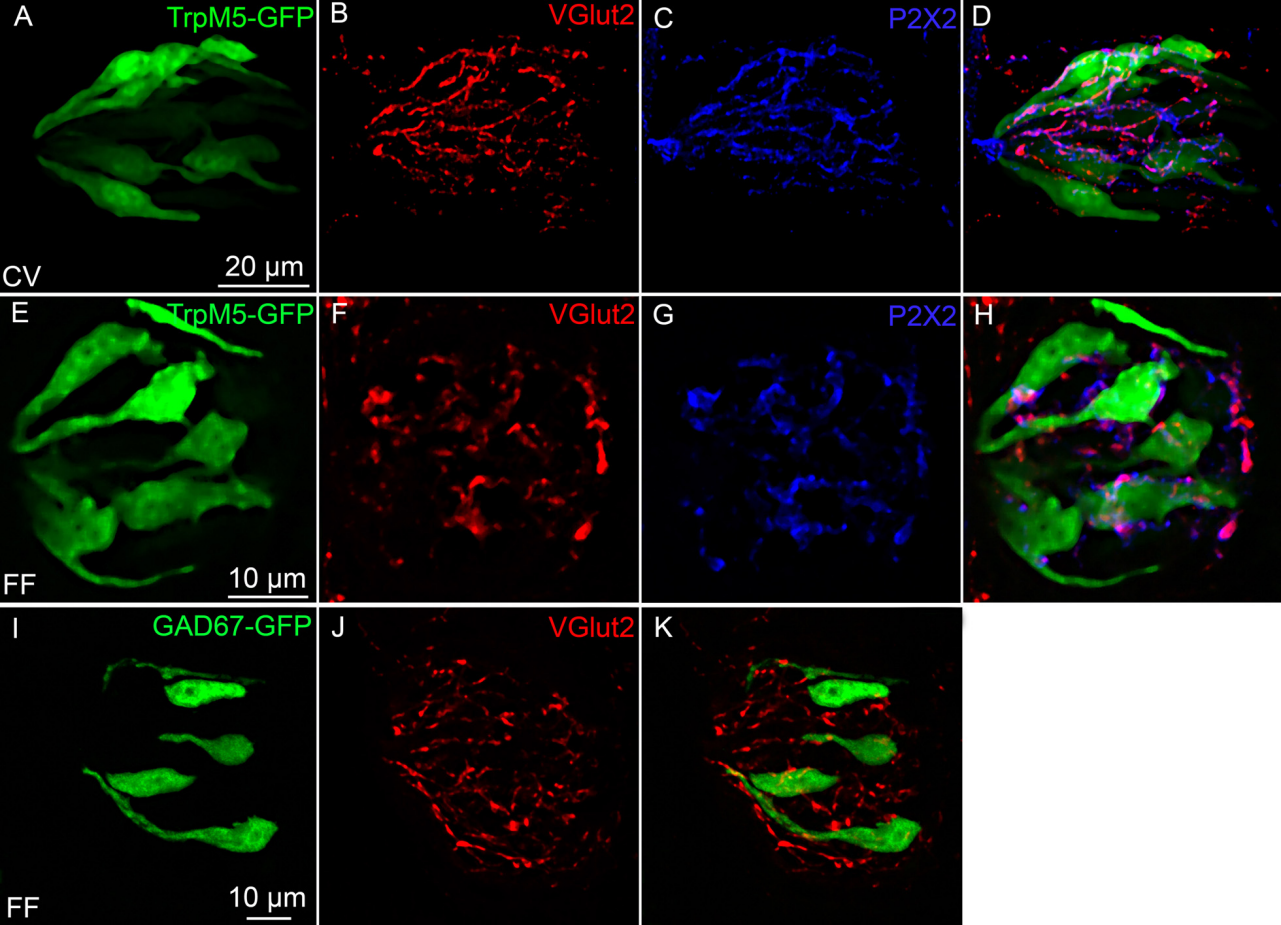
**VGLUT2 immunoreactivity in TRPM5-GFP and GAD67-GFP mice**. Laser scanning confocal images illustrate the expression of GFP under the control of the TRPM5 promoter (green), VGLUT2 (red) and P2X2 (blue) in circumvallate papillae (CV; A-D), fungiform papillae (FF; E-H). Panels I-K represent VGLUT2 immunoreactivity in GAD67-GFP mice. In fungiform and circumvallate taste buds, VGLUT2 is not expressed in taste cells but only in nerve fibers surrounding these taste cells. Moreover, the VGLUT2-positive fibers are co-localized with P2X2 fibers, a marker for gustatory fibers. Each figure represents merged images from a Z-series.

**Figure 4:**
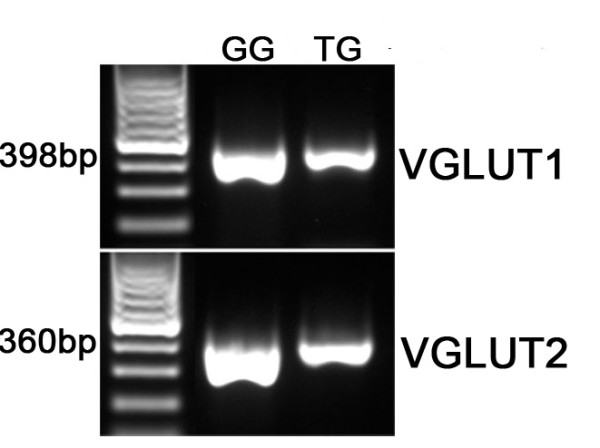
**Gel image of PCR products for VGLUT1 and 2 in geniculate and trigeminal ganglia**. The geniculate ganglion (GG) and the trigeminal ganglion (TG) both expressed VGLUT1 and 2.

**Figure 5:**
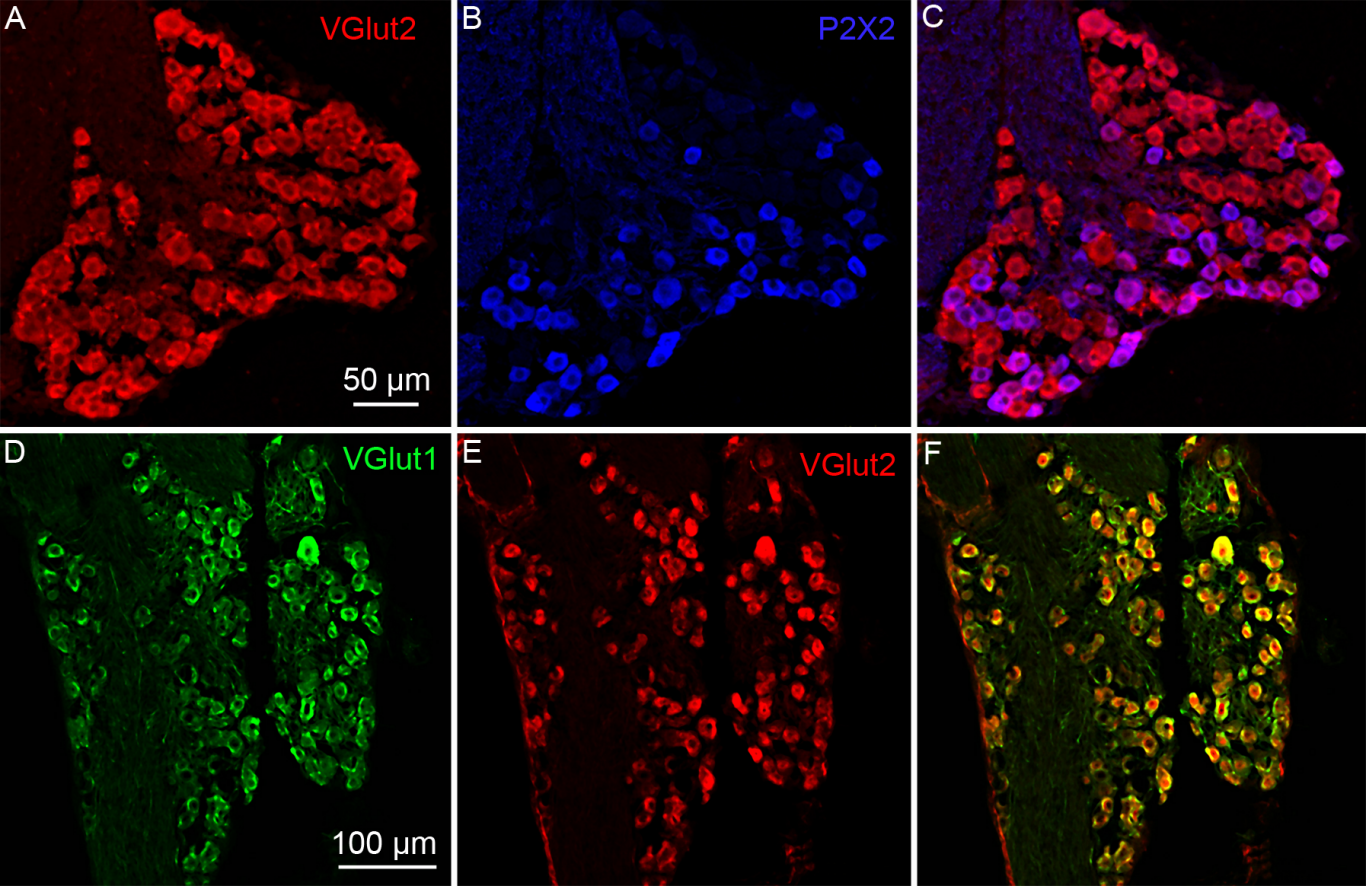
**VGLUT1 and 2 immunoreactivity in geniculate ganglion neurons**. A-C: Laser scanning photomicrographs of VGLUT2 (red) and P2X2 (blue). D-F: VGLUT1 (green), VGLUT2 (red). Note that all P2X2-expressing neurons also express VGLUT2 and that VGLUT2 completely co-localizes with VGLUT1.

Glutamate is usually released via a vesicular mechanism, with the uptake of glutamate into vesicles regulated by vesicular glutamate transporters (VGLUTs) [32]. We extracted mRNA from pooled taste buds isolated from circumvallate (CV) and fungiform (FF) papillae and used RT-PCR to determine if VGLUTs are expressed in taste cells. No RT-PCR products were detected for VGLUTs 1, 2, or 3 in the isolated taste buds, although products of the correct size were expressed in brain (Figure 1). Moreover, immunohistochemistry using specific antibodies against the VGLUTs did not label any taste cells (Figures 2 and 3). However, sections of circumvallate and fungiform papillae show that both VGLUT1 and VGLUT2 immunoreactivity are heavily expressed on nerve fibers innervating taste buds (Figures 2 and 3). No specific immunoreactivity for VGLUT3 was observed in taste cells or nerve fibers (data not shown). Since taste cells are surrounded by somatosensory as well as gustatory nerve fibers, we used an antibody against P2X2, a specific marker of gustatory nerve fibers [36] to determine whether the VGLUT2 antibody specifically labels gustatory nerve fibers. The double labeling showed that VGLUT2 is co-expressed with P2X2; i.e., all P2X2 fibers were immunoreactive for VGLUT2, but a few VGLUT2 positive fibers did not label with the P2X2 antibody (Figure 3). This result suggests that the majority of VGLUT2-labeled fibers are gustatory fibers. To confirm this, we isolated mRNA from the geniculate ganglion, which contains the cell bodies of the chorda tympani nerve fibers that innervate the fungiform taste buds. RT-PCR showed the expected expression of VGLUT1 and 2 in the ganglion (Figure 4). In addition, the ganglion was also sectioned and stained with antibodies against VGLUT1 and 2, as well as P2X2. The results confirmed that the majority of ganglion cells were immunoreactive for both VGLUT1 and 2 and that VGLUT2-immunoreactive cells also co-expressed P2X2 (Figure 5). We also examined co-localization of VGLUTs 1 and 2 in the ganglion, since in most systems these two VGLUTs are expressed in mutually exclusive neuronal populations [37]). However, we found that nearly all geniculate ganglion cells co-expressed VGLUT1 and 2. Taken together, these results indicate that gustatory ganglion cells express VGLUTs and that these VGLUTs are targeted to the distal end of the fibers within taste buds.

### Taste cells respond to glutamate stimulation

**Figure 6:**
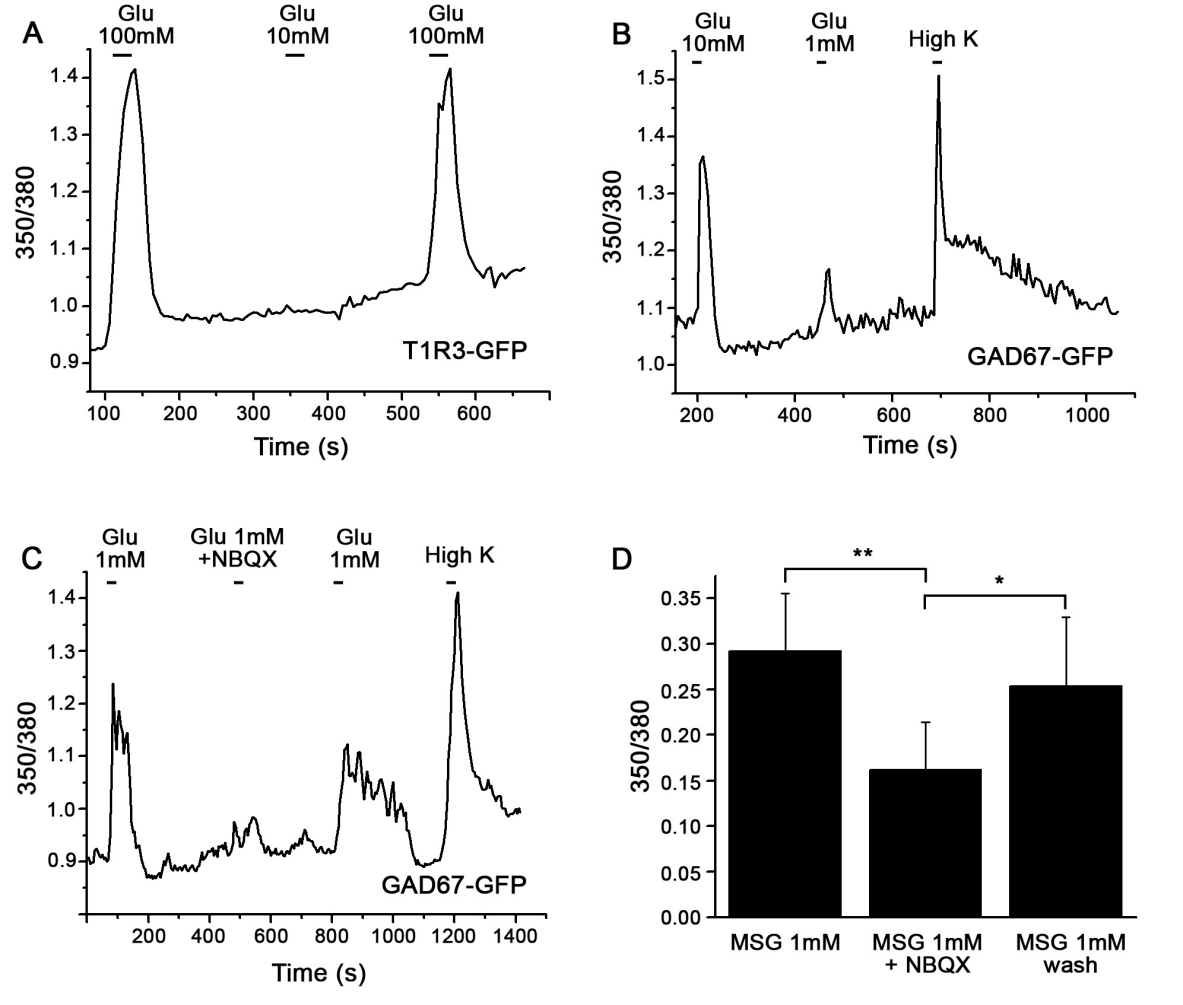
**Calcium responses to glutamate in Type II and Type III taste cells**. A: In T1R3-GFP cells, only high concentrations of glutamate (100 mM) elicit a calcium response. B: In GAD67-GFP cells, glutamate at 10 mM or 1 mM may elicit a calcium response. Type III cells possess voltage-gated calcium channels and respond to high concentrations of KCl (55 mM). C: In GAD67-GFP cells, NBQX, a specific antagonist of AMPA/Kainate receptors partially inhibited the response to 1 mM glutamate. D: Average effect of NBQX on responses to glutamate (One way repeated measures ANOVA with a Tukey post test; **: P < 0.005; *: P < 0.05, n = 9 cells).

VGLUT-labeled nerve fibers were present throughout the taste bud. Labeled fibers appeared to associate with both TRPM5-GFP labelled Type II cells (e.g., Figure 2 and 3) and GAD67-GFP labelled Type III cells (Figure 3). To further investigate which cell type might be the target of glutamate released from nerve fibers, we used calcium imaging to test whether Type II and/or Type III taste cells respond to glutamate (as monosodium glutamate) applied at concentrations expected for a neurotransmitter. Since taste cells may also respond to glutamate as a taste stimulus, we chose concentrations that would allow us to differentiate taste responses from putative neurotransmitter responses. For these experiments, 1 mM and 10 mM glutamate were used to elicit neurotransmitter responses, and 100 mM was used to activate glutamate taste receptors responsible for transducing umami taste [38]. Transgenic mice expressing GFP from the T1R3 promoter were used to identify a subset of Type II taste cells [21], those responding to umami and sweet taste stimuli, and mice expressing GFP from the GAD67 promoter to identify Type III cells, since GAD67 is expressed in a large subset of them [31]. In addition, cells were also stimulated with a 55 mM KCl solution. Only Type III cells express voltage-gated calcium channels and respond to the application of KCl with an increase of intracellular calcium [21]. T1R3-GFP cells responded primarily to 100 mM glutamate. A total of 6 fungiform T1R3-GFP cells (out of 46 cells tested), 2 circumvallate T1R3-GFP cells (out of 18) and 2 palate T1R3-GFP cells (out of 13) responded to glutamate at 100 mM (Figure 6A). Only 2 of these responding cells also responded to glutamate at 10 mM. In GAD67-GFP cells, out of 53 circumvallate cells tested, 15 responded to 1 mM glutamate while 14 additional cells responded to 10 mM glutamate (Figure 6B). All the GAD67-GFP cells showed a response to the high K^+ ^stimulation as expected. These results demonstrate a difference in the sensitivity of taste cells to glutamate, with Type III cells responding to concentrations likely to activate neurotransmitter receptors and Type II cells requiring a higher concentration of glutamate usually detected as a tastant.

### Glutamate receptors involved in the glutamate response

**Figure 7:**
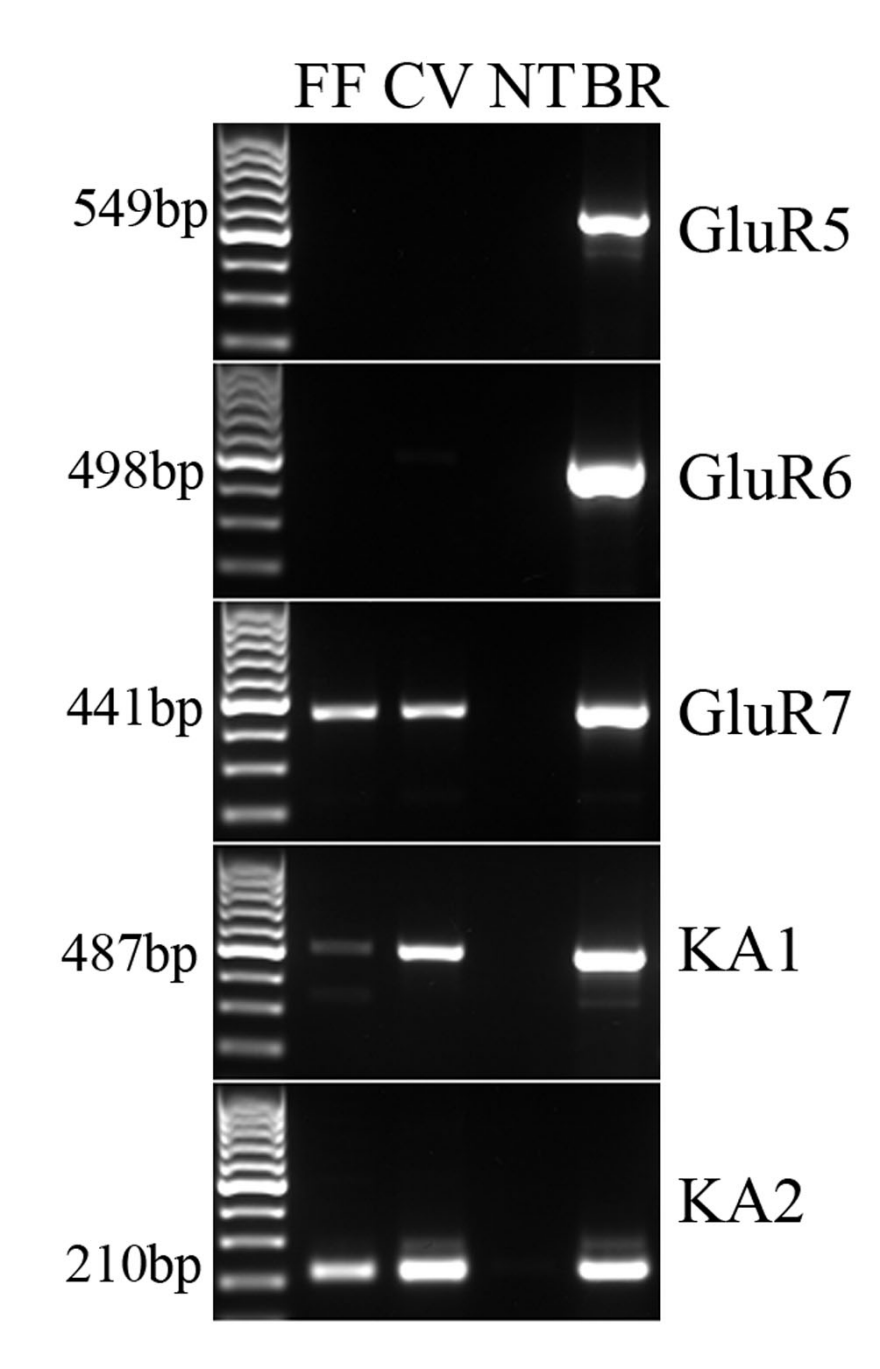
**Gel image of PCR products for Kainate receptors in taste tissues**. PCR products for GluR7, KA1 and KA2 were observed in pooled taste buds from fungiform (FF) and circumvallate (CV) papillae but not in non taste epithelium (NT). Brain (BR) was used as a control. Bands are for the expected size for GluR5 (549 bp), GluR6 (498 bp), GluR7 (441 bp), KA1 (487 bp) and KA2 (210 bp). Left lane indicates a 100-bp ladder.

**Figure 8:**
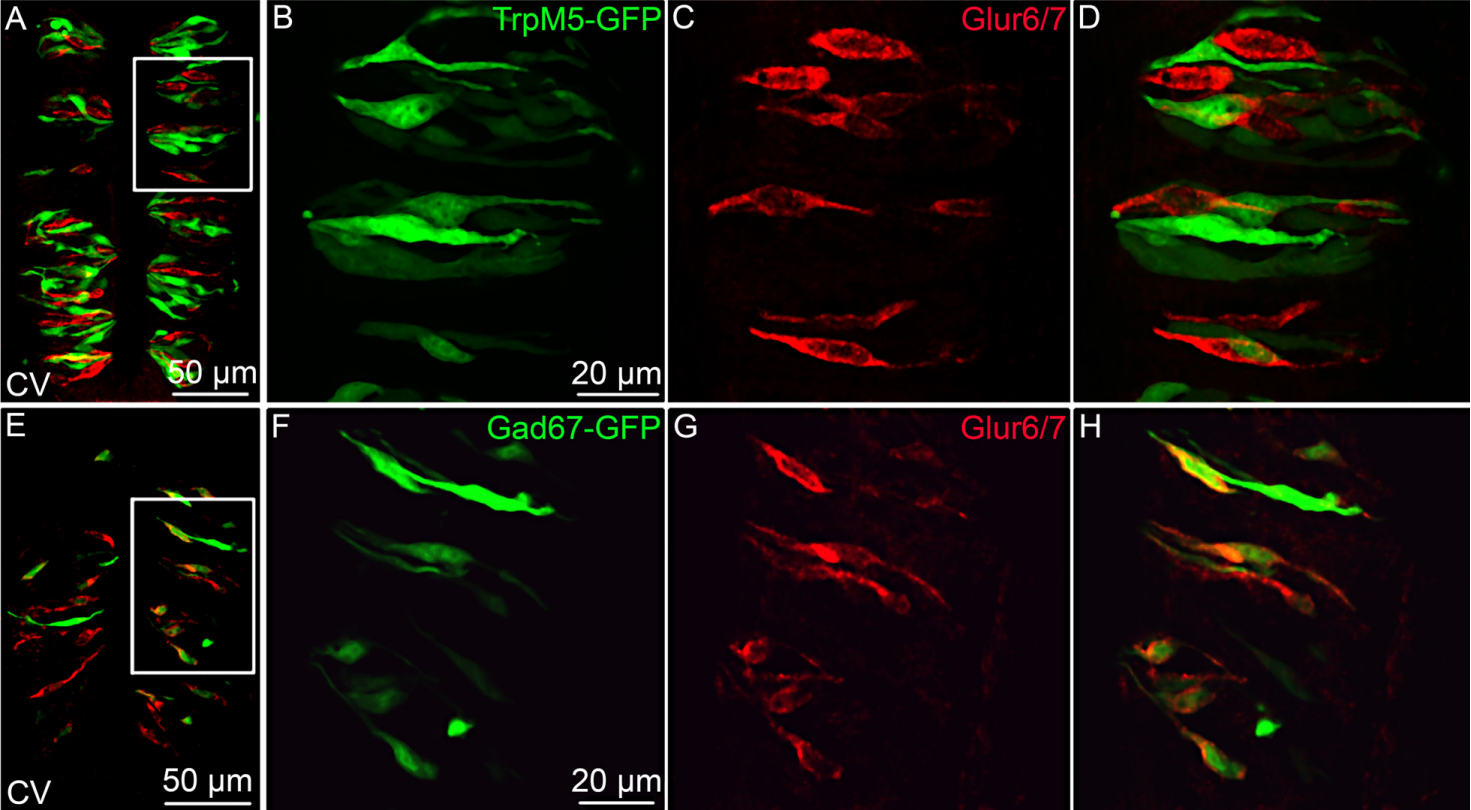
**GluR7 immunoreactivity in taste tissues of TRM5-GFP and GAD67-GFP mice**. The expression of GluR7 is shown in circumvallate (CV) papillae in mice expressing GFP (green) under the control of the TRPM5 promoter (A-D) or under the GAD67 promoter (E-H). The GluR7 is co-localized with a subset of GAD67-GFP cells but not with TRPM5-GFP cells suggesting that GluR7 is expressed in a subset of Type III cells. The overlay represents a maximum intensity projection obtained from a z series stack of images, so that the labelled taste cells are not in the same layer.

We used an AMPA/Kainate glutamate receptor antagonist, NBQX, to identify AMPA/Kainate ionotropic glutamate receptors involved in the response elicited by glutamate in Type III cells, since these cells responded to glutamate at concentrations expected for a neurotransmitter receptor. NBQX partially blocked the response to 1 mM glutamate (n = 9 cells) as shown in Figure 6C, D. Since previous studies have shown that taste cells fail to respond to AMPA agonists [4,15], we assumed that the receptor must be a member of the Kainate family, and we designed primers against GluR5-7 (now known as GLUK1-3), KA1 (now known as GLUK4), and KA2 (now knows as GLUK5). We used RT-PCR assays on mRNA isolated from pooled circumvallate taste buds, fungiform taste buds and non taste tissue. GluR7, and KA1 and KA2 were present in circumvallate and fungiform papillae (Figure 7). Non taste tissue was devoid of PCR products for these receptors. We used immunohistochemistry to identify the taste cells expressing GluR7, since KA1 and KA2 are usually associated with GluR7 to form functional receptors [39]. An antibody against GluR6/7 labeled subsets of taste cells in circumvallate, but not fungiform papillae. In all circumvallate taste buds examined, GluR7 co-localizes with a subset of GAD67-GFP cells but not with TRPM5 -GFP cells (Table 1, Figure 8). These data demonstrate that GluR7 is expressed specifically in Type III taste cells, which supports our results from calcium imaging studies.

**Table 1:** Number of cells expressing GluR7 in TRPM5-GFP and GAD67-GFP circumvallate taste buds

	Number of GFP cells	Number of GluR7 cells	Number of co-localized cells
TRPM5	295	161	0

GAD67	125	102	86

## Discussion

Our results suggest that the glutamate vesicular transporters, VGLUT1 and 2 are expressed in nerve fibers innervating taste buds but not in taste cells themselves. These data imply that afferent nerve fibers, rather than taste cells have the machinery to release glutamate into the taste bud to potentially modulate the activity of taste cells. Further, we report physiological, molecular, and immunohistochemical evidence that one likely target of the glutamate is the Kainate receptor GluR7, which is localized in Type III cells.

Somatosensory nerve fibers originating from the trigeminal ganglion also penetrate taste buds, and their cell bodies express VGLUT1 and 2 isoforms e.g, Figure. 4[40]. To differentiate the origin of the VGLUT-positive fibers (trigeminal or gustatory), the P2X2 antibody was used as a marker for gustatory fibers, since cell bodies in the trigeminal ganglion lack P2X2 expression [36]. Our results show that the majority of VGLUT2 immunoreactive nerve fibers in taste buds also express P2X2, suggesting a gustatory origin. We confirmed this by examining expression of VGLUTs in the geniculate ganglion, where the majority of the cells innervate taste buds on the tongue and palate. Most VGLUT2-expressing geniculate cell bodies also expressed P2X2, confirming that gustatory fibers express VGLUT2. Our data confirm those of a preliminary study showing expression of VGLUT1 and 2 but not 3 in nerve fibers associated with taste buds in mice [41]. However, another study recently reported immunohistochemical evidence for VGLUT expression in the taste tissue of rats, but only VGLUT1 was detected [42]. Further studies will be required to determine if this is a species difference.

To determine which cell types would respond to glutamate at concentrations expected for a neurotransmitter, we performed calcium imaging studies on GFP-labeled Type II and Type III taste cells. GAD67-GFP Type III cells showed responses to glutamate at concentrations of 10 mM (55% of the stimulated cells) or 1 mM (48% of the stimulated cells). In contrast, T1R3-GFP Type II cells failed to respond to 1 mM glutamate and only 2 out of 77 cells responded to 10 mM glutamate. However, several Type II cells responded to 100 mM glutamate, which is the appropriate concentration to activate the umami taste receptor, T1R1-T1R3 (isolated taste cells: [38]; chorda tympani recordings: [13]). These results strongly suggest that the neurotransmitter receptors for glutamate likely reside in the Type III cells rather than the Type II cells. However, we cannot exclude the possibility that other Type II cells that do not express T1R3 may respond to low concentrations of glutamate. Caicedo et al. [4] showed that the responses to 1 mM glutamate are blocked by CNQX, a specific AMPA/Kainate antagonist. When injected into the lingual artery, CNQX also decreased taste responses of the chorda tympani [43]. Our physiological experiments using NBQX, another AMPA/Kainate antagonist, confirm a role for AMPA/Kainate receptors in Type III mouse taste cells. However, the NBQX block was not complete, suggesting other receptors may participate in the glutamate response of Type III cells, as suggested by other investigators [44].One possibility is the NMDA receptor, previously identified in physiological studies of rat taste cells [45,46]. Although our data suggest that the neurotransmitter receptors are restricted to Type III taste cells, we cannot rule out the possible role of mGluRs in Type II taste cells that signal through the cAMP signaling cascade. Metabotropic glutamate receptors belonging to Class II (mGluR 2 and 3) and Class III (mGluR4) elicit decreases in cAMP [47], which would not be detected in a Ca^2+ ^imaging assay, since Type II taste cells lack voltage-gated Ca^2+ ^channels [21,31]. In addition, we would not be able to detect the presence of ionotropic GluRs that are not calcium permeable. However, NMDA receptors and kainate receptors are all Ca^2+ ^permeable, as are AMPA receptors lacking GluR2 [48]. We were not able to identify GluR2 in pooled RNA from circumvallate papillae (data not shown), although some immunoreactivity was found for GluR2/3 in rat circumvallate taste buds [49].

Using RT-PCR performed on pooled taste buds from fungiform and circumvallate papillae, and immunohistochemistry on sections of circumvallate taste tissue, we have now identified specific Kainate receptors involved in the glutamate response of Type III cells. RT-PCR products for GluR7, KA1 and KA2 were detected only in taste papillae and immunohistochemistry showed that an antibody directed against GluR6/7 specifically labels a subset of Type III taste cells. Since GluR6 was not detected in taste buds by RT-PCR, GluR7 is the likely GluR expressed in these taste cells. In the Kainate family of iGluRs, the GluR5-7 subunits constitute the low-affinity subunits and the KA1 and KA2 subunits constitute the high affinity subunits. The KA1 and KA2 subunits fail to form a functional homomeric receptor but create a functional channel when they form heteromeric receptors with GluR5-7 (for a review, see [39]). Interestingly, in HEK cells transfected with homomeric GluR7 or heteromeric GluR7/KA1 or GluR7/KA2, the EC50 value in response to glutamate application is in the 6 mM range [50], which is consistent with the levels of glutamate required to activate Type III cells in our physiological experiments. GluR7 has been studied in hippocampal mossy fiber synapses where it forms heteromeric receptors with GluR6 and KA1 and KA2. As in taste cells, it forms a low affinity, Ca^2+ ^permeable channel where, when activated, it modulates the release of the presynaptic transmitter [51].

The presence of VGLUTs in gustatory nerve fibers innervating taste cells clearly indicates that glutamate is stored in vesicles in the close vicinity of taste cells, where it could modulate the activity of Type III taste cells via activation of GluR7. Interestingly, omega shaped images of clear vesicles, likely representing exocytotic release of transmitter, have been observed in nerve fibers abutting taste buds [52]. Type II "receptor" cells respond to taste stimulation by release of ATP, which activates purinergic receptors on afferent nerve fibers [23] and adjacent taste cells [24,53]. In response, Type III cells release 5-HT, norepinephrine and possibly additional transmitters [30,53] to modulate Type II cells and activate nerve fibers, although the identity of the transmitter at the afferent synapse between Type III taste cells and nerve fibers is not known. These transmitters may activate the VGLUT-expressing afferent nerve fibers, which in turn would release glutamate to selectively modulate Type III taste cells. Excess glutamate and ATP would then be removed from the taste bud via transporters [9] and degradative enzymes [20] expressed in Type I taste cells. The role of the glutamate modulation is unclear, but Type III cells are the only taste cells that are responsive to multiple taste stimuli [54,55]. We speculate that if glutamate is released from nerve fibers, this VGLUT-mediated circuit may participate in the broad tuning of the Type III taste cells as well as to modulate the release of the Type III cell transmitters.

## Conclusions

The principal finding in this study is that gustatory afferent nerve fibers, rather than taste receptor cells, possess the machinery for vesicular release of glutamate in taste buds. Further, we show that a potential target of glutamate released from nerve fibers is the Kainate receptor GluR7, expressed selectively in a subset of Type III taste cells. We speculate that glutamate may be involved in a signalling circuit in taste buds to modulate transmitter release from Type III taste cells.

## Methods

### Animals

All experimental procedures were approved by the Animal Care and Use Committees of the UC Denver School of Medicine and Colorado State University. Adult transgenic mice in which either the TRPM5 or the T1R3 promoter drives the expression of Green Fluorescent Protein (GFP) were used to identify Type II taste cells (both in C57/BL6 background). Breeding pairs were provided by Robert Margolskee and expression of GFP in Type II taste cells was validated by Clapp et al. [21]. Mice expressing GFP from the GAD67 promoter (in C57/BL6 background) were used to identify Type III taste cells. These mice were purchased from Jackson Laboratory (Bar Harbor, ME) and the expression of GFP in taste tissue was validated by DeFazio et al. [31]. For RT-PCR experiments, C57/BL6 mice were used.

### Solutions

Tyrode's solution contained the following (in mM): 140 NaCl, 5 KCl, 1 MgCl_2_, 2 CaCl_2_, 10 HEPES, 10 glucose, and 1 Na pyruvate, adjusted to pH 7.4 with NaOH. High K^+ ^solution was used to discriminate Type III cells from other cell types and contained the following (in mM): 90 NaCl, 55 KCl, 1 MgCl, 1 CaCl_2_, 10 HEPES, 10 glucose, and 1 Na pyruvate. Ca^2+^/Mg^2+ ^free Tyrode's solution contained 1 mM BAPTA. Monosodium glutamate (1 mM, 10 mM and 100 mM, Sigma, St. Louis, MO) was used as a stimulus. The concentration of NaCl in Tyrode's was adjusted in these solutions in order to maintain the initial 140 mM concentration of Na^+^. A specific AMPA/Kainate receptor antagonist (2,3-Dioxo-6-nitro-1,2,3,4-tetrahydrobenzoquinoxaline-7-sulfonamide disodium salt or NBQX) was used at the concentration of 16-40 μM and mixed with 1 mM glutamate.

### Taste cell isolation

Circumvallate taste cells were isolated using the protocol of Béhé et al. [56]. Briefly, mice were killed with CO_2 _and cervical dislocation. An enzyme cocktail consisting of 0.7 mg/ml collagenase B (Roche, Indianapolis, IN), 3 mg/ml Dispase II (Roche, Indianapolis, IN), and 1 mg/ml trypsin inhibitor (Sigma, St. Louis, MO) dissolved in Tyrode's was injected beneath the epithelium of the tongue. After incubation for 40 minutes in Ca^2+ ^free Tyrode's, the epithelium was gently separated from the underlying connective tissue and placed in Ca^2+ ^free Tyrode's containing 1 mM BAPTA for 10 min. Taste buds were removed by gentle suction with a fire-polished pipette and placed in an Eppendorf tube containing 200 μl RNAlater (Qiagen, Valencia, CA) for the RT-PCR experiments, or plated onto cover slips coated with poly-L-lysine (Sigma, St. Louis, MO) for the calcium imaging experiments.

The geniculate and trigeminal ganglia were dissected from surrounding tissues and placed in RNAlater for the RT-PCR experiments or in fixative for the immunohistochemistry experiments.

### RT-PCR

RNA pooled from isolated fungiform and circumvallate taste buds was extracted according to manufacturer's instructions using the RNeasy Micro kit from Qiagen (Valencia, CA), including a two hour DNase I treatment at room temperature for removal of genomic DNA. Reverse transcription was performed using the iScript cDNA Synthesis kit from Biorad (Hercules, CA). Control reactions lacking the reverse transcriptase enzyme were used to detect potential DNA contamination. Two microliters of cDNA were added to the PCR reaction (Qiagen Taq PCR Core kit, Valencia, CA). PCR conditions included an initial 5 min. denaturation step followed by 35 cycles of 30s denaturation at 95°C, 30s annealing at 60°C, and 45s extension at 72°C; concluding with a 7 min. final extension step. To validate the PCR, we included cDNA from whole brain (Clontech, Mountain View, CA), non-taste lingual epithelium and a no template control (water). The non-taste lingual epithelium was removed from the area surrounding the circumvallate papillae. This tissue did not express α-gustducin or PLCβ2 in RT-PCR, in contrast to taste tissue. Amplified sequences were visualized by gel electrophoresis in 1% agarose gels stained with GelRed (Biotium, Hayward, CA). Experiments were repeated three independent times. PCR products were purified with the Qiagen QIAquick PCR Purification kit or QIAEX II Gel Extraction kit (Valencia, CA) and sequences were determined using an ABI 3730 DNA Sequencer (Foster City, CA) in the UCDenver sequencing core. Sequences were compared with those published using NCBI BLAST. Primers listed in Table 2 were designed using IDT PrimerQuest and spanned at least one intron to avoid amplification of genomic DNA.

**Table 2:** Accession number, DNA sequence, and product sizes of primers for Kainate receptor subunits and VGLUTs.

Gene	Accession #	Forward Primer (5'→3')	Reverse Primer (5'→3')	Product (bp)
GluR5	NM_010348	TGCCTAGGAGTCAGTTGTGTGCTT	AGTGAGGTTGCAGTTCCTCTGTGT	549

GluR6	NM_010349	GTGCCATCTTGGATTTGGTGCAGT	ACATCAGAGCAGCATCAGTCGTCA	498

GluR7	NM_001081097	TCACACAGAGGAACTGCAACCTCA	TGATAACCGCATCCGTCTTGACCA	441

KA1	NM_175481	TGCTTCCTGCTTGGCTCTTGAT	AGGCATTCTGCTTTGGCACA	487

KA2	NM_008168	TGATGCCAATGCGTCCATCT	ATGTTGAGGCTGCGCACAAA	210

VGlut1	NM_182993	ACCTGTTCTGGTTGCTTGTCTCCT	GTTCATGAGCTTTCGCACGTTGGT	398

VGlut2	NM_080853	AGCCTGTCATGGGATATGGAGCAA	ATTATCGCGTAGACGGGCATGGAT	360

VGlut3	NM_182959	TCACATCCTTGCCTGTCTATGCCA	TGAGGAACACATTCTGCCACTCCT	525

### Calcium Imaging

Isolated cells or small clusters of cells were used. Intracellular Ca^2+ ^measurements were obtained after loading taste cells with ~2 μM fura-2 AM (Molecular Probes, Invitrogen Corporation), as described previously [21]. Images were acquired with the CCD Sensicam QE camera (COOKE Corporation) through a 40× oil immersion objective lens of an inverted Nikon Diaphot TMD microscope. Excitation wavelengths of 350 nm and 380 nm were used with an emission wavelength ~510 nm. Calcium levels were reported as F350/F380 versus time. Images were captured every 5 seconds using Imaging Workbench 5.2 (Indec Biosystems, Inc.). All solutions were bath applied using a gravity flow perfusion system (Automate Scientific Inc., San Francisco, CA) and laminar flow perfusion chambers (RC-25F, Warner Scientific Inc., Hamden, CT).

### Immunohistochemistry

Mice were perfusion-fixed in 4% paraformaldehyde and 0.1 M Phosphate Buffer or in periodate-lysine-paraformaldehyde (PLP) fixative that contained 75 mM lysine, 10 mM sodium periodate, and 1.6% paraformaldehyde. Tissues were postfixed (4°C, 3 hours) and cryoprotected in 20% sucrose-phosphate buffer (4°C, overnight). Fourteen μm cryosections were collected and dried onto FisherPlus slides overnight, then washed with phosphate-buffered saline (PBS), blocked with 2% normal goat or donkey serum before incubation in primary antibody. The specific primary antibodies used are listed in Table 3. The secondary antiserum used was Alexa 568 goat anti-rabbit IgG (1:400; Molecular Probe, Invitrogen, CA) for GluR6/7 and VGLUT1, Alexa 568 goat anti-guinea pig (1:400; Molecular Probe, Invitrogen, CA) for VGLUT2 and VGLUT3 and DyeLight 647 donkey anti-rabbit (1:400, Jackson ImmunoResearch Laboratories, PA) for P2X2. To control for the non-specificity of the secondary antibody, the primary antibodies were omitted in some sections; no reactivity was observed in these preparations. As a control for the expression of VGLUT3, we used VGLUT3 knock out mice [33]. Any expression that was present in both knockout and wildtype mice was considered to be nonspecific. All primary antibodies have been previously validated in other studies (Table 3). All images were collected with an Olympus Fluoview confocal laser scanning microscope (LSCM) FV300 (Olympus Corporation). For each image, the channels were collected sequentially with single-wavelength excitation and then merged to produce the composite image using the software Fluoview 5.0. Images were adjusted for brightness and gamma using Photoshop 7. For some images, as indicated in the captions, digital filters (Photoshop Dust & Scratches, Unsharp Mask) were used to eliminate pixel noise and to enhance local contrast values.

**Table 3:** Catalog number, dilution, source, immunogen and reference for the primary antibodies used

Antibody (catalog number)	Rabbit anti-rat GluR6/7 (04-921)	Rabbit anti-rat VGLUT1 (135303)	Guinea pig anti-rat VGLUT2 (AB2251)	Rabbit anti-rat P2X2 (ARR-003)	Guinea pig anti-rat VGLUT3 (AB5421)
**Dilution**	1:100	1:500/1:1000	1:2000	1:1000	1:2000

**Source**	Millipore, MA	Synaptic systems, Germany	Millipore, MA	Alomone Labs, Israel	Millipore, MA

**Immunogen**	894-908	456-560	17aa in C-terminal region	457-472	

**Reference**	[57]	[58]	[59]	[60]	[61]

## Authors' contributions

Immunohistochemistry was done by AV, MT, LMS and DB. CBA carried out the RT-PCR. AV carried out the calcium imaging experiments. AV, LMS and SCK designed the experiments. AV and SCK wrote the manuscript. All authors read and approved the manuscript.
